# LINE-1 hypomethylation is associated with poor outcomes in locoregionally advanced oropharyngeal cancer

**DOI:** 10.1186/s13148-022-01386-5

**Published:** 2022-12-12

**Authors:** Mariateresa Casarotto, Valentina Lupato, Giorgio Giurato, Roberto Guerrieri, Sandro Sulfaro, Annamaria Salvati, Elisa D’Angelo, Carlo Furlan, Anna Menegaldo, Lorena Baboci, Barbara Montico, Irene Turturici, Riccardo Dolcetti, Salvatore Romeo, Vittorio Baggio, Stefania Corrado, Gianluca Businello, Maria Guido, Alessandro Weisz, Vittorio Giacomarra, Giovanni Franchin, Agostino Steffan, Luca Sigalotti, Emanuela Vaccher, Paolo Boscolo-Rizzo, Polesel Jerry, Giuseppe Fanetti, Elisabetta Fratta

**Affiliations:** 1 Unit of Immunopathology and Cancer Biomarkers, Centro di Riferimento Oncologico di Aviano (CRO), IRCCS, Aviano, Italy; 2 Division of Otolaryngology, General Hospital “S. Maria Degli Angeli”, Pordenone, Italy; 3 Laboratory of Molecular Medicine and Genomics, Department of Medicine, Surgery and Dentistry ‘Scuola Medica Salernitana’, University of Salerno, Baronissi, SA Italy; 4 Genome Research Center for Health, Campus of Medicine, University of Salerno, Baronissi, SA Italy; 5 Division of Pathology, General Hospital “S. Maria Degli Angeli”, Pordenone, Italy; 6 Medical Genomics Program, AOU ‘SS. Giovanni di Dio e Ruggi d’Aragona’, University of Salerno, Salerno, Italy; 7 Department of Radiation Oncology, University Hospital of Modena, Modena, Italy; 8 Department of Radiation Oncology, General Hospital “San Martino”, Belluno, Italy; 9 Unit of Otolaryngology, AULSS 2 - Marca Trevigiana, Treviso, Italy; 10 Division of Radiotherapy, Centro di Riferimento Oncologico di Aviano (CRO), IRCCS, Aviano, PN Italy; 11 Peter MacCallum Cancer Centre, Melbourne, VIC 3000 Australia; 12 Sir Peter MacCallum Department of Oncology, The University of Melbourne, Melbourne, VIC 3010 Australia; 13 Department of Microbiology and Immunology, The University of Melbourne, Melbourne, VIC 3010 Australia; 14 Department of Services of Diagnosis and Care, Santorso Hospital, Santorso, VI Italy; 15 Department of Radiation Oncology, Treviso Regional Hospital, Treviso, Italy; 16 Department of Anatomy and Pathology, University Hospital of Modena, Modena, Italy; 17 Department of Pathology, Treviso Regional Hospital, Treviso, Italy; 18 Department of Medicine (DIMED), University of Padova, Padova, Italy; 19 Oncogenetics and Functional Oncogenomics Unit, Centro di Riferimento Oncologico di Aviano (CRO), IRCCS, Aviano, Italy; 20 Division of Medical Oncology A, Centro di Riferimento Oncologico di Aviano (CRO), IRCCS, Aviano, Italy; 21 Section of Otolaryngology, Department of Neurosciences, University of Padova, Treviso, Italy; 22 Unit of Cancer Epidemiology, Centro di Riferimento Oncologico di Aviano (CRO), IRCCS, Aviano, Italy; 23 Division of Immunopathology and Cancer Biomarkers, Centro di Riferimento Oncologico di Aviano (CRO), IRCCS, Via Franco Gallini, 2, 33081 Aviano, PN Italy

**Keywords:** Oropharyngeal squamous cell carcinoma, HPV, LINE-1, DNA methylation, p53

## Abstract

**Background and purpose:**

Currently, human papillomavirus (HPV) positivity represents a strong prognostic factor for both reduced risk of relapse and improved survival in patients with oropharyngeal squamous cell carcinoma (OPSCC). However, a subset of HPV-positive OPSCC patients still experience poor outcomes. Furthermore, HPV-negative OPSCC patients, who have an even higher risk of relapse, are still lacking suitable prognostic biomarkers for clinical outcome. Here, we evaluated the prognostic value of LINE-1 methylation level in OPSCC patients and further addressed the relationship between LINE-1 methylation status and p53 protein expression as well as genome-wide/gene-specific DNA methylation.

**Results:**

In this study, DNA was extracted from 163 formalin-fixed paraffin-embedded tissue samples retrospectively collected from stage III-IVB OPSCC patients managed with curative intent with up-front treatment. Quantitative methylation-specific PCR revealed that LINE-1 hypomethylation was directly associated with poor prognosis (5-year overall survival—OS: 28.1% for LINE-1 methylation < 35% vs. 69.1% for ≥ 55%; *p* < 0.0001). When LINE-1 methylation was dichotomized as < 55% versus ≥ 55%, interaction with HPV16 emerged: compared with hypermethylated HPV16-positive patients, subjects with hypomethylated HPV16-negative OPSCC reported an adjusted higher risk of death (HR 4.83, 95% CI 2.24–10.38) and progression (HR 4.54, 95% CI 2.18–9.48). Tumor protein p53 (*TP53*) gene is often mutated and overexpressed in HPV-negative OPSCC. Since p53 has been reported to repress LINE-1 promoter, we then analyzed the association between p53 protein expression and LINE-1 methylation levels. Following p53 immunohistochemistry, results indicated that among HPV16-negative patients with p53 ≥ 50%, LINE-1 methylation levels declined and remained stable at approximately 43%; any HPV16-positive patient reported p53 ≥ 50%. Finally, DNA methylation analysis demonstrated that genome-wide average methylation level at cytosine–phosphate–guanine sites was significantly lower in HPV16-negative OPSCC patients who relapsed within two years. The subsequent integrative analysis of gene expression and DNA methylation identified 20 up-regulated/hypomethylated genes in relapsed patients, and most of them contained LINE-1 elements in their promoter sequences.

**Conclusions:**

Evaluation of the methylation level of LINE-1 may help in identifying the subset of OPSCC patients with bad prognosis regardless of their HPV status. Aberrant LINE-1 hypomethylation might occur along with *TP53* mutations and lead to altered gene expression in OPSCC.

**Supplementary Information:**

The online version contains supplementary material available at 10.1186/s13148-022-01386-5.

## Introduction

Changes in sexual habits have led to a steady increase in the incidence of human papillomavirus (HPV)-driven oropharyngeal squamous cell carcinoma (OPSCC) that in most Western countries now exceeds the proportion of tobacco- and alcohol-related counterparts [1]. Of note, HPV16 represents the most common genotype (83%) among HPV-positive OPSCC patients [2]. Hence, HPV16 is by far the most carcinogenic HPV type in OPSCC, as most non-HPV16 oncogenic infections do not progress to cancer [3]. Although HPV confers a substantial survival benefit to these malignancies [4], roughly 20% of all patients with HPV-positive OPSCC develop recurrent disease within 5 years after diagnosis [5–8]. Given the considerable interest in identifying treatment de-escalation strategies in this subset of OPSCC patients with a more favorable prognosis [9], it is of paramount importance to identify biological predictors of atypical behavior to avoid the administration of sub-optimal treatment.

Genes encoding for epigenetic regulators have been frequently found mutated in several tumors, including OPSCC [10]; hence, it is expected that epigenetic changes may play an important role in OPSCC pathogenesis and response to therapy. Consistently, HPV oncoproteins have also been demonstrated to impact on the epigenetic patterns by interacting with different epigenetic regulators [11], thus affecting the chromatin landscape of HPV-positive OPSCC cells. At present, DNA methylation represents one of the most investigated epigenetic mechanisms along with histone modifications and non-coding RNAs. DNA methylation consists of the addition of a methyl group to the fifth carbon of cytosines residues within cytosine–phosphate–guanine (CpG) sites. It has been estimated that over 90% of these CpG sites are located within DNA repetitive elements, particularly Alu and LINE-1 [12]. LINE-1 is the most abundant repetitive element in the genome, which in normal human tissues is generally found heavily methylated. Since it represents approximately 17% of the human genome, LINE-1 has been widely accepted as a surrogate marker of global DNA methylation [13]. In the last years, LINE-1 hypomethylation has been showed to be related to carcinogenesis and to the development of many tumor types [14–17]. On these grounds, we previously observed a significantly lower level of LINE-1 methylation in OPSCC patients who relapsed within 2 years, thus indicating that the overall level of genomic DNA methylation may have an impact on the risk of early relapse in OPSCC [18]. Despite these findings, however, the prognostic impact of LINE-1 methylation levels on OPSCC survival has not been established.

Hypomethylation in the LINE-1 promoter region is crucial for the transcriptional activation of LINE-1 elements, which results in retroelement transposition and genomic instability, thus providing a setting for cancer progression [19]. In the last years, it has become increasingly clear that several transcription factors and chromatin remodelers are involved in LINE-1 activation [20, 21]. Among these, a number of studies have suggested that p53 protein might silence LINE-1 through regulating the deposition of epigenetic marks within its promoter [22–25], thus affecting its retrotransposon activity in tumor cells. Notably, somatic mutations of the tumor suppressor gene *TP53* are one of the most frequent alterations in head and neck squamous cell carcinoma (HNSCC) [26]. Besides mutations, p53 functions can also be disrupted by the HPV E6 protein in HPV-positive patients [27]. At present, it is largely unknown whether *TP53* mutational status and/or p53 expression pattern correlates with LINE-1 methylation in OPSCC.

About 30% of the transcription start sites in the human genome are associated with repetitive elements, particularly with LINE-1 subfamilies [28]; furthermore, following loss of methylation, LINE-1 was shown to act as an alternative promoter for surrounding genes [29, 30]. Therefore, another aspect yet to be investigated is how the OPSCC epigenome evolves in relapsed patients respect to non-relapsed ones and whether the differentially methylated regions between the two subgroups map within LINE-1 elements.

Based on these premises, this study aimed to assess the impact of LINE-1 methylation level on overall survival (OS) and progression-free survival (PFS) in both HPV-positive and HPV-negative OPSCC patients. In addition, to better determine whether p53 expression might affect LINE-1 methylation status in OPSCC, we evaluated the correlation between p53 expression pattern and LINE-1 methylation levels. Finally, to shed initial light on the mechanisms through which LINE1 methylation impacts OPSCC outcome, differences in genome-wide/gene-specific DNA methylation were investigated in a subset of relapsed and not relapsed OPSCC patients and further investigated for their potential regulation by LINE-1 elements.

## Methods

### Patients

The study enrolled 163 stage III-IVB OPSCC patients managed with curative intent with up-front (chemo-)radiotherapy or up-front surgery followed by adjuvant (chemo-)radiotherapy, as previously described [18]. Patients have been treated between 2001 and 2019 at the National Cancer Institute in Aviano, the “Santa Maria degli Angeli” General Hospital in Pordenone, the “Ca’ Foncello” General Hospital in Treviso, and the University Hospital in Modena. All tumors were centrally reclassified according to the American Joint Committee on Cancer 7th Edition. The study was approved by the local Independent Ethic Committees (CRO-2019-13, 733/AULSS2, 5/2020/OSS/AOUMO). Participants provided written informed consent for inclusion in the study; 104 subjects overlapped with a previous study [18].

### Immunohistochemical analysis of p53

For each patient, we retrieved a formalin-fixed paraffin-embedded (FFPE) tissues representative of the OPSCC, collected at the time of biopsy or surgical resection and before starting any treatment, for a total of 163 neoplastic samples. Serial 5-µm-thick FFPE tumor sections were then used for hematoxylin—eosin staining and immunohistochemistry (IHC) analysis. All stained sections were microscopically evaluated by a pathologist unaware of any clinical information (including follow-up or outcome data), and only neoplastic lesions that contained at least 70% of neoplastic cells were included in the study.

p53 expression was evaluated by IHC (Agilent Technologies DAKO; Clone DO-7) in 89 patients, for whom sufficient neoplastic sample was available. The extent of staining was estimated to the nearest 10% level of positive tumor cells. The intensity of staining was recorded as weak or strong. Strong expression in more than 50% of cells or complete absence of stain was considered a p53 mutated pattern [31–35].

### Quantitation of HPV16 E6 DNA using real-time quantitative PCR analysis

Genomic DNA was extracted from OPSCC FFPE tissues using the FFPE RNA/DNA Purification Plus Kit (Norgen), following the manufacturer’s protocol. SYBR green quantitative HPV16-PCR was carried out as previously reported [18].

### Quantitative methylation-specific PCR analysis for the methylation levels of LINE-1

Genomic DNA was obtained from OPSCC FFPE tissues in quantities sufficient for bisulfite treatment. Bisulfite conversion was carried out on 500 ng genomic DNA using EZ DNA Methylation-Gold™ Kit (Zymo Research), according to the manufacturer’s protocol. SYBR Green quantitative methylation-specific PCR (qMSP) was performed as previously reported [18].

### Genome-wide DNA methylation analysis

DNA methylation analysis was performed by Genomix4Life S.R.L. (Baronissi, Salerno, Italy). To assess the quality of DNA isolated by FFPE samples, Illumina FFPE QC kit (Illumina, San Diego, CA, USA) was used. Only 10 FFPE DNA samples were considered eligible for restoration using the Infinium HD FFPE Restore Kit (Illumina, San Diego, CA, USA). Restored DNA was bisulfite converted using EZ DNA methylation kit (Zymo Research). For each sample, 250 ng of bisulfite converted DNA was used for analysis of whole-genome methylation using MethylationEPIC BeadChip (Illumina, San Diego, CA, USA), which contains 850,000 probes. In brief, bisulfite-converted DNA was whole-genome amplified for 20 h followed by end-point fragmentation. Fragmented DNA was precipitated, denaturated, and hybridized to the BeadChips for 20 h at 48 °C. The BeadChips were washed, and the hybridized primers were extended and labeled before scanning the BeadChips using the Illumina iScan system. GenomeStudio software was used for the extraction of DNA methylation signals from scanned arrays.

### RNA extraction and transcriptome profiling

RNA isolations were performed from 5 FFPE samples using the FFPE RNA/DNA Purification Plus Kit (Norgen). Nucleic acids were quantified with Qubit 2.0 fluorimeter using Qubit RNA HS assay kit (Termo Fisher Scientifc, USA), and the assessment of nucleic acids integrity (RNA Integrity Number) was performed with Agilent 4150 TapeStation System (Agilent Technologies, USA). Only 5 samples passed the qualitative and quantitative checks required by the Illumina library protocol. Libraries preparation for transcriptome analysis was performed employing the TruSeq RNA Exome kit (Cat.20020189, Illumina) for FFPE samples starting from 200 ng of RNA as input materials, respectively, according to manufacturers’protocols; 5 libraries were sequenced on NextSeq 500 (Illumina) using 2 × 75pb paired end.

### Bioinformatics analysis

EPIC methylation array was performed using ChAMP [33]. Only CpG with a detection *p* < 0.01 was considered for further analysis. The analysis was performed by comparing patients who relapsed within 2 years from the end of treatment with those who did not, and only the CpG associated with a *p* < 0.05 and DeltaBeta (DB) cutoff set to the first quartile value (|DB|≥ 0.15) of DB distribution were considered differentially methylated. Genomic annotation of CpGs was performed using the information available in Infinium MethylationEPIC v1.0 B5 Manifest file. In detail, transcriptional start site (TSS)200 refers to CpGs between 0 and 200 bases upstream of the TSS; TSS1500 refers to CpGs between 200 and 1500 bases upstream of the TSS; 5’UTR refers to those GpGs within the 5’ untranslated region, between the TSS and the ATG start site; gene body refers to CpGs between the ATG and stop codon, regardless of the presence of introns and exons. Promoter region includes TSS1500, TSS200, 5’UTR, and 1st exon regions. Annotation of LINE on selected genes was performed using the Genome Browser track “Repeating Elements by RepeatMasker” [34]. Functional analysis on potentially up- and down-regulated genes was performed using IPA (Ingenuity Pathway Analysis, Qiagen). Only molecular functions with a *p* ≤ 0.05 were considered.

RNA-Seq data analysis was performed as previously described [35]. In detail, quality control of sequenced reads has been performed using FASTQC (https://www.bioinformatics.babraham.ac.uk/projects/fastqc/), while adapter sequences were removed using Trimmomatic [36]. Alignment was performed on human genome (assembly hg38) considering GenCode Release 41 (GRCh38.p13) with STAR v2.7.10a [37], setting the default parameters. Quantification of expressed transcripts was performed using FeatureCounts [38] and differentially expressed transcripts were identified using DESeq2 [39]. Differential expression was performed on relapsed vs relapse-free OPSCC patients. Differentially expressed transcripts were reported as |Fold-Change|(FC) ≥ 1.5 along with associated adjusted *p* ≤ 0.05, computed according to Benjamini-Hochberg.

### Statistical methods

Distribution of patients according to sociodemographic and clinical characteristics was reported as absolute number and corresponding percentage. LINE-1 methylation was reported as median value with interquartile range (IQR). Differences between strata were evaluated through Kruskal–Wallis test. Further, to evaluate associations between LINE-1 methylation, HPV status, and p53 expression, the analysis of variance was conducted, with *post hoc* Tukey test.

LINE-1 methylation was then categorized in three levels (< 35%, 35–54%, and ≥ 55%) using a recursive procedure which identifies the cutoffs which maximize the difference in OS. The optimal cutoffs were in agreement with previous findings [18]. For each patient, the time at risk was calculated from date of elective treatment completion to the event of interest or last follow-up, whichever came first. The event of interest was death (any cause) for OS and death or locoregional/distant recurrence for PFS. The Kaplan–Meier method was used to generate crude survival probabilities, and the log-rank test was used to assess the difference in time to event according to LINE-1 methylation level and HPV16 status [36]. To account for potential confounders, hazard ratios (HRs) and the corresponding 95% confidence intervals (CIs) were calculated using Cox proportional hazards models [36], adjusting for gender and age, plus covariates significantly associated with OS in the univariate analysis (i.e., T stage, N stage, and HPV16 status).

## Results

### Prognostic impact of LINE-1 methylation

**Fig. 1:**
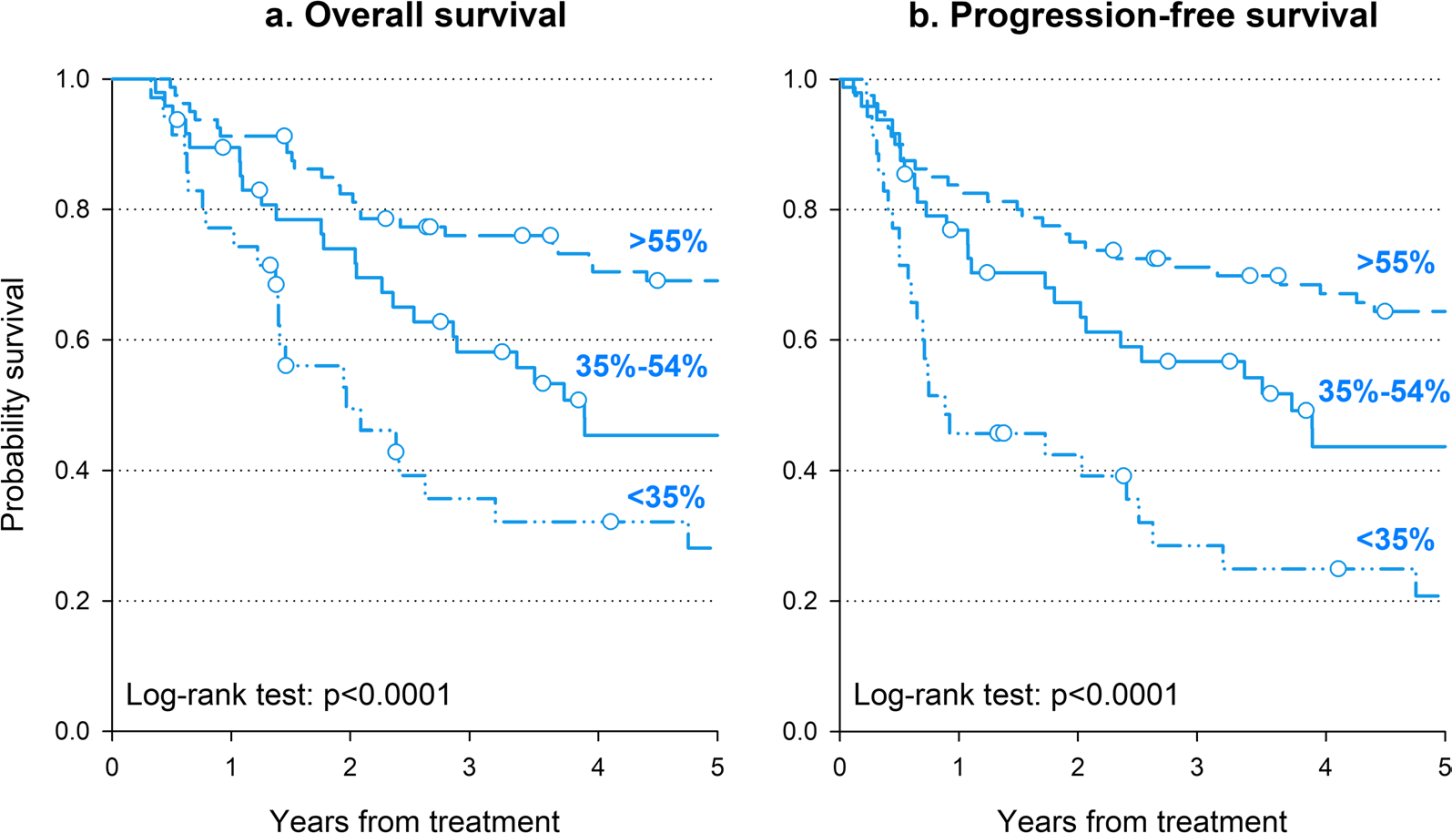
Oncological outcomes according to level of LINE-1 methylation. Overall survival (**a**) and progression-free survival (**b**) were calculated through the Kaplan–Meier method. LINE-1 methylation was categorized using a recursive procedure which maximizes the difference in OS

Table 1 shows the median LINE-1 expression according to sociodemographic and clinical characteristics in 163 OPSCC patients. Majority of the patients were males (71.8%), with TNM stage IVA-B (76.7%) and HPV16-negative (67.5%); 90 patients (55.2%) underwent surgery. LINE-1 expression was lower in patients aged ≥ 70 years than younger ones (*p* = 0.0249) and in HPV16-negative than in HPV16-positive patients (*p* < 0.0001). LINE-1 methylation level was directly associated with prognosis, with survival rates decreasing with LINE-1 hypomethylation (Fig. 1). In detail, patients with LINE-1 methylation ≥ 55% reported a 5-year OS of 69.1% compared to 45.5% for LINE-1 methylation between 35 and 54%, and to 28.1% for LINE-1 methylation < 35% (Fig. 1a, *p* < 0.0001). Similarly, 5-year PFS probabilities were 64.4%, 43.7%, and 20.8%, for LINE-1 methylation ≥ 55%, between 35 and 54%, and < 35%, respectively (Fig. 1b, *p* < 0.0001). Multivariate analyses confirmed that patients with LINE-1 < 35% had a worse prognosis than those with LINE-1 ≥ 55% (Table 2), with a HR of 2.76 (95% CI 1.48–5.12) for death and of 2.39 (95% CI 1.35–4.24) for progression/death. Interestingly, excess risk in patients with LINE-1 < 35% remained significant after adjustment for HPV16 status. Patients with LINE-1 35–54% were at increased risk of both death and progression/death, but the HRs were no longer statistically significant.

**Table 1:** LINE-1 methylation in 163 patients with stage III-IV oropharyngeal squamous cell carcinoma according to sociodemographic and clinical characteristics

	Patients		LINE-1 (%)	
	*N*	(%)	Median (Q1–Q3)	
Sex				
Man	117	(71.8)	53.7 (38.5–72.7)	*p* = 0.8192
Woman	46	(28.2)	55.8 (38.1–70.6)	
Age (years)				
< 60	40	(24.5)	59.6 (38.6–75.3)	*p* = 0.0249
60–69	57	(35.0)	61.3 (42.2–73.0)	
≥ 70	66	(40.5)	47.8 (24.1–59.3)	
T stage^a^				
T1	24	(14.7)	70.0 (44.8–77.8)	*p* = 0.1137
T2	51	(31.3)	61.8 (41.0–75.5)	
T3	57	(35.0)	51.0 (30.2–65.0)	
T4	31	(19.0)	53.4 (38.6–75.3)	
N stage^a^				
N0	17	(10.4)	51.0 (20.0–66.2)	*p* = 0.2205
N1	28	(17.2)	50.0 (34.2–74.4)	
N2	105	(64.4)	59.7 (41.9–73.0)	
N3	13	(8.0)	52.2 (30.3–69.5)	
Stage^a^				
III	38	(23.3)	48.9 (27.1–70.1)	*p* = 0.1475
IV	125	(76.7)	58.2 (41.0–72.7)	
HPV-status				
Negative	110	(67.5)	50.1 (30.3–66.2)	*p* < 0.0001^b^
Positive	46	(28.2)	71.2 (58.6–78.2)	
Unknown	7	(4.3)	46.1 (29.5–51.1)	
Surgery				
No	73	(44.8)	58.6 (42.1–77.2)	*p* = 0.1144
Yes	90	(55.2)	52.5 (37.9–70.1)	

^a^TNM staging according to the American Joint Committee on Cancer 7th Edition.

^b^Excluding missing values

**Table 2:** Hazard ratio (HR) and corresponding 95% confidence interval (CI) for progression-free survival and overall survival in 163 patients with stage III–IV oropharyngeal squamous cell carcinoma

	Patients	Overall survival			Progression-free survival		
		Events	HR (95% CI)^a^	HR (95% CI)^b^	Events	HR (95% CI)^a^	HR (95% CI)^b^
Sex							
Man	117	64	Reference	Reference	69	Reference	Reference
Woman	46	26	1.03 (0.64–1.63)	1.05 (0.65–1.70)	29	1.20 (0.77–1.87)	1.28 (0.81–2.02)
Age (years)							
< 60	40	20	Reference	Reference	22	Reference	Reference
60–69	57	26	1.00 (0.54–1.83)	1.06 (0.57–1.98)	31	1.11 (0.63–1.97)	1.18 (0.66–2.15)
≥ 70	66	44	1.91 (1.10–3.33)	1.47 (0.83–2.64)	45	1.80 (1.05–3.07)	1.54 (0.88–2.58)
T stage^c^							
T1-T2	75	35	Reference	Reference	37	Reference	Reference
T3-T4	88	55	1.74 (1.13–2.69)	1.42 (0.89–2.25)	61	1.76 (1.16–2.68)	1.32 (0.84–2.08)
N stage^c^							
N0-N1	45	29	Reference	Reference	30	Reference	Reference
N2	105	51	0.79 (0.50–1.26)	1.20 (0.71–2.02)	58	0.86 (0.55–1.34)	1.13 (0.69–1.85)
N3	13	10	2.29 (1.10–4.78)	2.87 (1.34–6.14)	10	2.29 (1.10–4.73)	2.65 (1.26–5.59)
HPV-status							
Negative	110	76	Reference	Reference	82	Reference	Reference
Positive	46	13	0.30 (0.16–0.54)	0.43 (0.23–0.81)	14	0.29 (0.16–0.51)	0.39 (0.21–0.72)
Surgery							
No	73	40	Reference		43	Reference	
Yes	90	50	1.17 (0.75–1.82)		55	1.08 (0.70–1.65)	
LINE-1 methylation							
≥ 55%	80	34	Reference	Reference	40	Reference	Reference
35–54%	48	28	1.60 (0.96–2.67)	1.46 (0.85–2.49)	29	1.41 (0.86–2.31)	1.25 (0.75–2.07)
< 35%	35	28	3.21 (1.89–5.45)	2.76 (1.48–5.12)	29	3.19 (1.92–5.30)	2.39 (1.35–4.24)

^a^Estimated from Cox proportional hazards model, adjusted for study center, sex, and age

^b^Further adjusted for T stage, N stage, HPV status, and LINE-1 methylation. ^c^TNM staging according to the American Joint Committee on Cancer 7th Edition

#### Association of LINE-1 methylation levels with HPV16 status

**Fig. 2:**
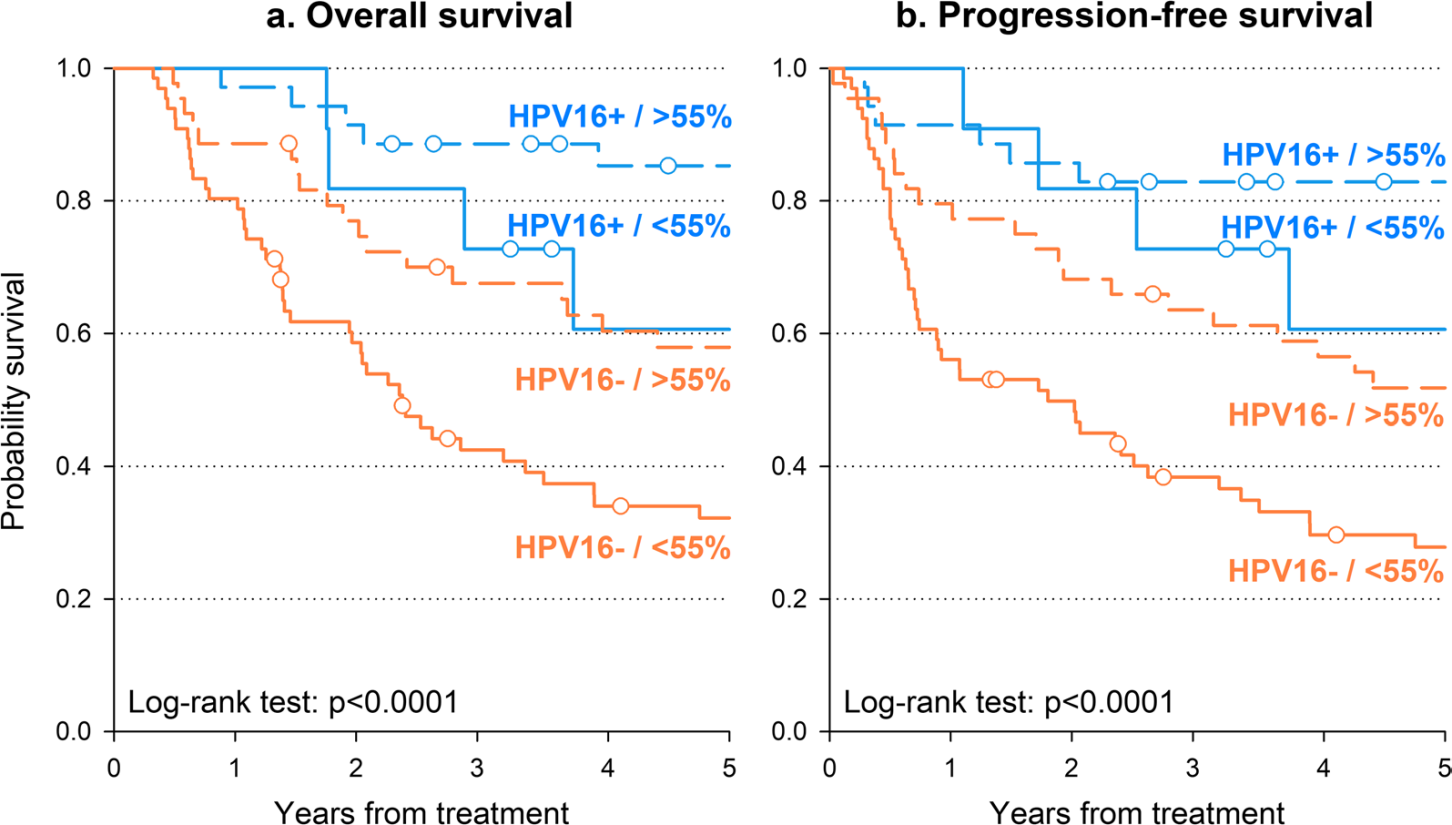
Oncological outcomes according to HPV16 status and level of LINE-1 methylation. Overall survival (**a**) and progression-free overall survival (**b**) were calculated through the Kaplan–Meier method, stratifying patients according to HPV16 status and LINE-1 methylation level

Potential interaction between HPV16 status and LINE-1 methylation levels was further investigated, dichotomizing LINE-1 as < 55% versus ≥ 55%. HPV16-positive patients with LINE-1 ≥ 55% showed the best 5-year OS (85.3%—Fig. 2a) and PFS (82.9%—Fig. 2b) in contrast with HPV16-negative patients with LINE-1 < 55% who reported the worst prognosis (32.2% and 27.8%, respectively). Interestingly, HPV16-positive patients with LINE-1 < 55% reported similar overall survival as HPV16-negative patients with LINE-1 ≥ 55%. These findings were confirmed by multivariable analyses (Additional file 1: Table S1), which showed a significantly increased risk of death or progression in HPV16-negative patients with LINE-1 < 55% compared to HPV16-positive patients with LINE-1 ≥ 55% (HR for death: 4.83, 95% CI 2.24–10.38; HR for death/progression: 4.54, 95% CI 2.18–9.48).

#### Association of LINE-1 methylation levels with p53 expression

**Fig. 3:**
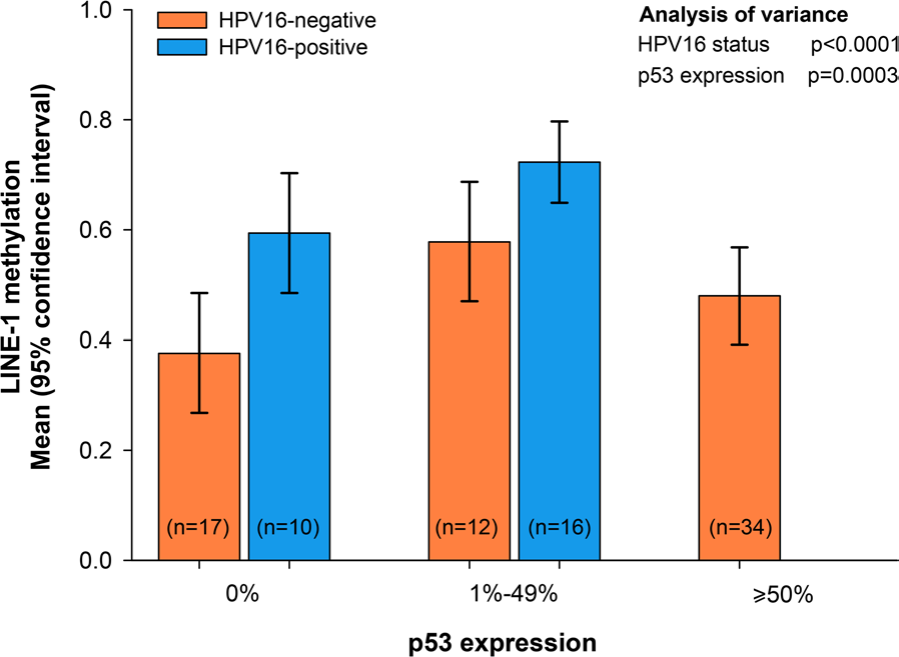
LINE-1 methylation according to HVP16 status and p53 expression pattern. Mean LINE-1 methylation, and corresponding 95% confidence intervals, was reported for HPV16 status and level of p53 expression. The independent association of HPV16 status and p53 expression with LINE-1 methylation was evaluated through the analysis of variance

Since p53 might control LINE-1 methylation, we then analyzed the association between p53 expression status and LINE-1 methylation levels in a sub-group of 89 patients (Additional file 2: Table S2). To this end, p53 expression pattern was categorized into three groups according to the overall intensity of nuclear staining of tumor cells and the extent of stained cells (i.e., 0%, 1–49%, ≥ 50%). Figure 3 shows mean LINE-1 methylation according to p53 expression and HPV16 status. For p53 expression < 50%, LINE-1 methylation increased with increasing p53 regardless of HPV16 status (*p* = 0.0003). Among HPV16-negative patients with p53 ≥ 50%, LINE-1 methylation levels declined and remained stable at approximately 43%. No HPV16-positive patients reported p53 ≥ 50%.

### Identification of differentially methylated CpG sites in relapsing patients

We recently reported a significant decrease in LINE-1 methylation in OPSCC patients who relapsed within 2 years from the end of treatment, especially in HPV16-negative ones [18]. Therefore, the methylation levels (beta-values) of CpG sites were analyzed in 5 HPV16-negative OPSCC patients who relapsed within 2 years and in 5 who did not, in order to investigate whether the differentially methylated regions between the two subgroups mapped within LINE-1 elements. Unfortunately, the sample size was limited due to the amount of genomic DNA required for the analysis (Additional file 2: Table S2).

**Fig. 4:**
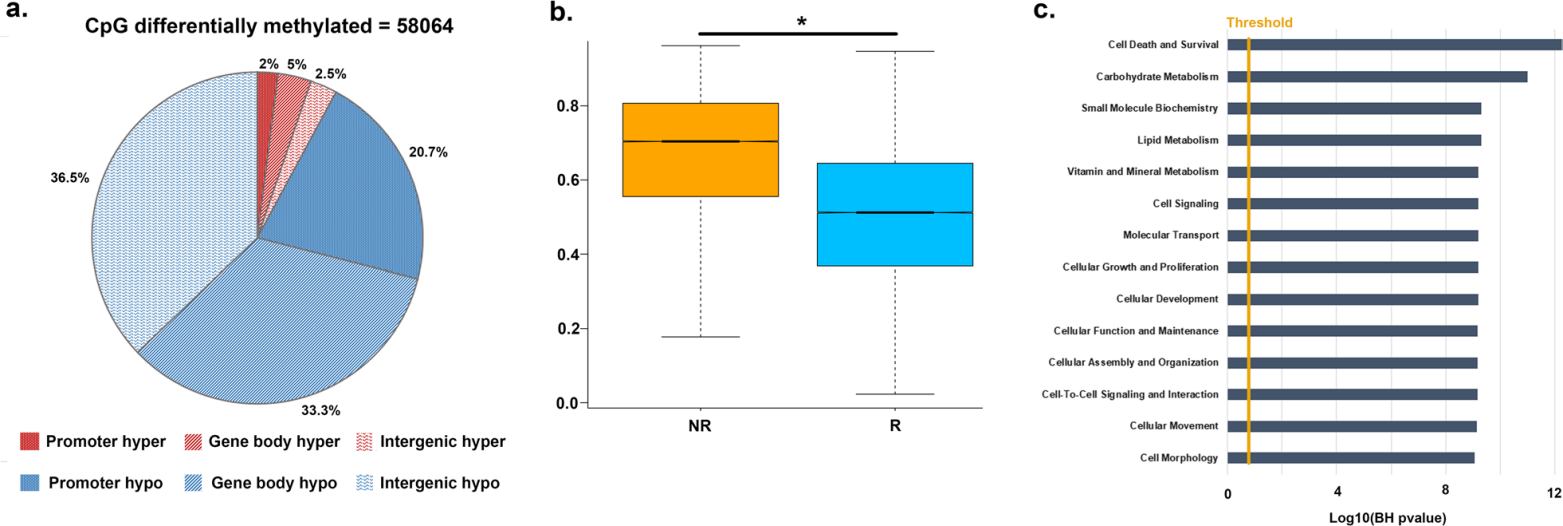
DNA methylation analysis in HPV16-negative OPSCC patients. **a** Pie chart showing the percentage of differentially methylated CpG in promoter, gene body, and intergenic regions. **b** Box plot showing the average methylation level (beta-value) of all CpG differentially methylated in not relapsed (NR) vs relapsed (R) HPV16-negative OPSCC patients. **c** Histogram showing the molecular function where the genes showing differentially methylated promoter are involved

Results indicated that there were 58,064 CpG (|DB|≥ 0.15 and *p* < 0.05) with a difference in the methylation level between OPSCC patients who relapsed compared to those who were relapse-free for at least 24 months after the treatment; in particular, 4500 CpG sites were hypermethylated, whereas 53,564 were hypomethylated (Fig. 4a). Therefore, a significantly lower content of CpG methylation could be found in relapsed (median value = 0.51) respect to relapse-free OPSCC patients (median value = 0.70) (Fig. 4b, Additional file 5: Figure S1). Among the 58,064 differentially methylated CpG, 38.3% overlapped with gene bodies, 39.0% were intergenic, whereas 22.7% overlapped with gene promoters (Fig. 4a, Additional file 3: Table S3). To identify DNA methylation alterations within the promoter regions, we focused on CpG sites located within the TSS1500, TSS200, 5’ UTR, and first exon. A global hypomethylation pattern was still observed since 12,036 of 17,819 CpGs were significantly hypomethylated in OPSCC patients who relapsed within 2 years. To further explore the biological roles of these CpGs, we performed gene ontology (GO) enrichment analysis. Results indicated that the most significantly enriched GO terms were molecular functions of potential importance for cancer development and progression, including cellular growth and proliferation, cell-to-cell signaling and interaction, cellular movement, and cell morphology (Fig. 4c). Notably, 3743 CpGs differentially methylated overlapped with LINE-1 elements (3502 hypomethylated and 241 hypermethylated).

### Correlation between CpG methylation and gene expression

**Fig. 5:**
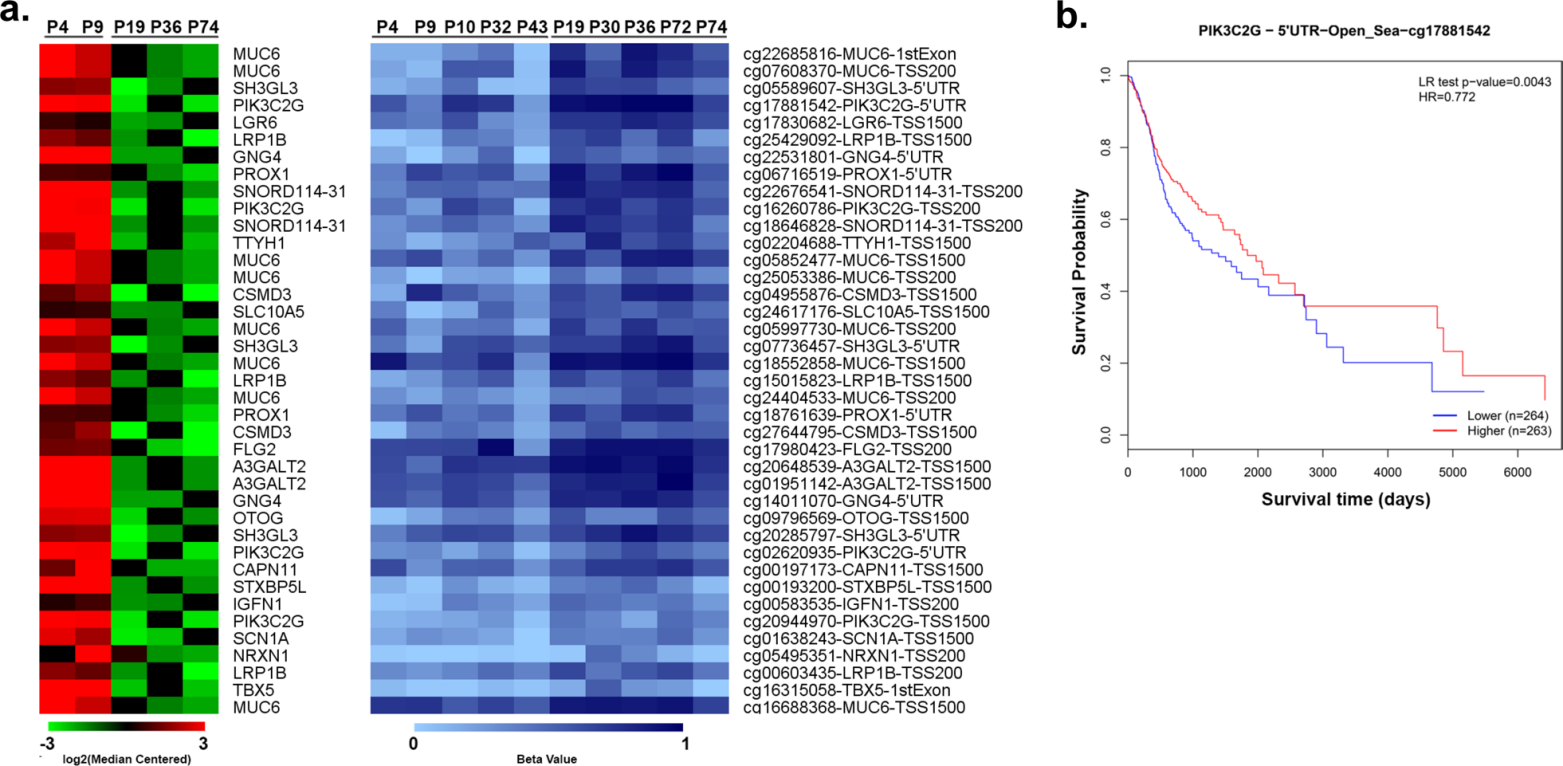
Integrative analysis of gene expression and DNA methylation in HPV16-negative OPSCC patients. **a** Leftside: heatmap summarizing expression data for the up-regulated genes in HPV16-negative OPSCC patients (P) who relapsed within 2 years from the end of treatment (P4, P9) with those who did not (P19, P36, P74). Data are shown as normalized expression values in log2 and centered on the median value. Rightside: heatmap showing the methylation values (beta value) in relapsed (P4, P9, P10, P32, P43) vs relapsed-free (P19, P30, P36, P72, P74) HPV16-negative OPSCC patients. **b** Kaplan–Meier estimates overall survival according to high- and low-methylation levels of the PIK3C2G cg17881542 in MethSurv

Aberrant DNA methylation patterns might contribute to differential survival through altered expression of the respective genes. Hence, gene expression profile was evaluated by RNA sequencing on 2 relapsed and 3 relapse-free HPV16-negative OPSCC patients, leading to the identification of 367 differentially expressed genes, of which 286 were down-regulated and 81 up-regulated (|FC|≥ 1.5 and adj-pval ≤ 0.05). Since it is generally accepted that promoter methylation is associated with decreased transcription of downstream genes and vice versa, gene expression and DNA methylation profiles were integrated to determine whether there were any connections. Setting |FC|≥ 1.5 and |Db|≥ 0.15 as cutoff, we identified 29 differentially expressed genes and 59 differentially methylated CpGs. We focused in particular on hypomethylated and up-regulated genes, thereby identifying 20 genes (Fig. 5a, Additional file 4: Table S4), most of which (16/20) overlapped with LINE-1 elements (Additional file 6: Figure S2–Additional file 9: Figure S5), with the exception of FLG2, MUC6, SLC10A5 and SNORD114-31 (Additional file 10: Fig. S6). These results suggest that LINE-1 hypomethylation might affect gene expression in OPSCC. Notably, a high number of LINE-1 elements were found within PIK3C2G, which represented one of the most hypomethylated and up-regulated genes in patients who early relapsed. Since PIK3C2G hypomethylation has been recently found to predict tumor relapse and shorter OS in ovarian cancer [37], the prognostic roles of PIK3C2G CpG in HNSCC were explored through the public database MethSurv (https://biit.cs.ut.ee/methsurv/) [38]. Only PIK3C2G cg17881542 was present in this dataset and, although the data were of borderline significance (log-rank test, *p* = 0.004; HR = 0.77, 95% CI 0.59–1.01), we could observe that high methylation levels of cg17881542 were associated with favorable prognosis in HNSCC (Fig. 5b).

## Discussion

In the last years, accumulating evidence indicated that loss of LINE-1 methylation is crucially involved in carcinogenesis; in fact, LINE-1 demethylation was found to promote genomic and chromosomal instabilities [39] and to activate the transcription of cancer-related genes as well [29, 30]. In addition, the epigenetic status of LINE-1 has been widely associated with patient outcomes in several malignancies (for review see [14]). In this context, we have recently demonstrated that LINE-1 methylation levels were lower in OPSCC patients who relapsed within 24 months [18], thus indicating that the overall level of genomic DNA methylation might have an impact on early OPSCC relapse risk. Consistently, this study demonstrated that hypomethylation of LINE-1 correlated with significantly poorer PFS and OS in an expanded retrospective cohort of 163 OPSCC patients. Although stratified survival analyses highlighted the prognostic significance of LINE-1 hypomethylation in OPSCC patients irrespective of HPV16 status, the lowest level of LINE-1 element methylation was observed in HPV16-negative tumors. Collectively, these results corroborate the finding that LINE-1 hypomethylation may be an effective biomarker to predict OPSCC survival and further suggest that epigenetic changes could overall contribute to OPSCC biology and could be partially responsible for the biological and clinical differences between HPV16-positive and HPV16-negative OPSCC patients. Measuring LINE-1 methylation levels at diagnosis may aid the clinician to schedule the frequency of follow-up examination and/or to choose the aggressiveness of treatment, especially in HPV-negative OPSCC patients. Importantly, global hypomethylation could allow rapidly proliferating and highly mutated tumors to escape immune reaction and to become resistant to immunotherapy [40]. Therefore, LINE-1 hypomethylation may also represent an independent indicator of poor immunotherapy responses in HPV-negative OPSCC tumors.

A recent study of de Carvalho et al. has shown that HPV-negative OPSCC tumors usually have a high mutation burden respect to HPV-positive ones [41]. In particular, *TP53* mutations are frequently found in OPSCC driven by alcohol and tobacco, whereas their presence has been reported in only a small subset of HPV-related OPSCC so far [42–46]. Unfortunately, in our study, OPSCC patients were not investigated for *TP53* mutation by sequencing analysis. However, since the complete absence of immunolabeling or IHC overexpression for p53 (≥ 50% positive cells) has been found to closely correlate with the presence of *TP53* mutations in several tumor types [31–35], p53 protein expression, as determined by IHC, was used as surrogate for *TP53* mutation status. In fact, according to several studies, *TP53* missense mutations resulted in nuclear accumulation and p53 overexpression, whereas absence of p53 staining was associated with nucleotide deletions or non-sense mutations that resulted in protein truncation. On the other hand, tumors with wild-type *TP53* displayed intermediate immunolabeling patterns [31–35]. When we evaluated the association between LINE-1 methylation status and p53 expression, we observed that p53 absence or a strong and diffuse pattern of p53 expression correlated with lower LINE-1 methylation levels in HPV16-negative OPSCC patients, whereas no p53 overexpression was found in patients with HPV16 infection, which is consistent with the mechanism of p53 degradation by HPV16 E6 [47]. Chromatin immunoprecipitation studies indicated extensive p53 enrichment within LINE-1 promoter region of the retrotransposon element LINE-1, thus suggesting that p53 might directly bind and recruit a variety of epigenetic regulators (i.e., DNA methyltransferases) in order to silence retroelements [25]. Hence, it seems plausible that aberrant LINE-1 hypomethylation may occur along with *TP53* mutations. Consistently, an increased expression of the LINE-1 retrotransposable element ORF1 protein has often been correlated with *TP53* mutations and aberrant p53 expression [23, 48, 49].

DNA methylation analysis demonstrated that genome-wide average methylation level at CpGs was significantly lower in OPSCC patients who relapsed within two years, thus confirming the important role played by DNA hypomethylation in OPSCC progression. Although the sample size included was limited, our data suggested that the methylation status of *PIK3C2G* gene might have particular relevance in OPSCC since it appeared to be strictly associated with LINE-1 elements (Additional file 8: Figure S4). Notably, the protein encoded by *PIK3C2G* represents a key extracellular signaling molecule participating in the PI3K/Akt signaling pathway. Activation of this pathway has shown to contribute to the development of resistance to chemotherapy and radiotherapy in several cancers, including HNSCC [50]. Consistently, HNSCC have shown mutations in more than one PI3K pathway molecule, including PIK3C2G [51]. Of interest, by using GeneMANIA (https://genemania.org/) [52], we found that PIK3C2G is co-expressed with GNG4 and NRXN1 (Additional file 11: Figure S7) which were among the 20 hypomethylated/up-regulated genes, and contained several LINE-1 elements within their promoters as well. GNG4 is a member of the G-protein family and, similar to PIK3C2G, has been closely associated with PI3K/Akt signaling pathway in HNSCC [53]. Although GNG4 has been reported to be hypermethylated and down-regulated in bladder cancer [54], breast cancer [55], and glioblastoma [56], other studies have shown that GNG4 expression was significantly up-regulated in lung carcinoma [57], and colorectal cancer [58]. More interestingly, in a paper by You et al., GNG4 has been listed as one of the up-regulated genes potentially involved in radioresistance in HNSCC [53]. NRXN1 represents a single-pass transmembrane protein and has been recently described as a potential novel target for antibody–drug conjugate therapy in small cell lung cancer [59]. Of note, NRXN1 was indicated as hypomethylated and overexpressed in HPV-positive HNSCC [60], thus suggesting that the role of NRXN1 should be better elucidated in the two OPSCC subtypes.

Despite these findings, our study has some limitations. First, this study was carried out on a retrospective cohort. Second, the detection of HPV in our samples was restricted to HPV16, the most common high-risk subtype associated with OPSCC, whereas less common subtypes (i.e., HPV18, HPV31, HPV33 and HPV52) were not evaluated. However, although the clinical behavior and pathogenesis of non-HPV16-OPSCC are less well known, recent studies indicated that the survival benefit of HPV-positivity might be mainly attributed to HPV16 genotype in OPSCC [61], whereas OS among non-HPV16 was even poorer than for HPV-negative HNSCC patients [62]. Third, FFPE material was not sufficient to identify genetic alterations of the *TP53* gene and to perform genome-wide DNA methylation and RNA-seq analyses in all OPSCC patients. Fourth, the formaldehyde-induced DNA inter-strand crosslinks might interfere with bisulfite conversion [63], which is a critical step for the quantitative analysis of LINE-1 methylation. In fact, unconverted cytosines, if present, would lead to possible bias in qMSP analysis. Finally, since qMSP assay covers a limited number of CpG sites within the promoter region, the clinical value of the LINE-1 methylation status might be representative only of the genomic location analyzed [64].

## Conclusion

In conclusion, our results clearly indicated that LINE-1 hypomethylation was associated with poorer OS and PFS in OPSCC patients regardless of their HPV16 status. Intriguingly, genome-wide methylation analysis suggested that hypomethylation of LINE-1 elements might promote the transcription of genes that are potentially involved in OPSCC. At present, a prospective study is ongoing to validate the prognostic significance of LINE-1 methylation in a larger sample cohort of OPSCC patients. Future research is also needed to elucidate whether p53 may affect retrotransposon activity in OPSCC cells and to better understand whether LINE-1 activity plays a direct role in OPSCC progression.

## Supplementary Information

**Additional file 1: Table S1.** Hazard ratio (HR) and corresponding 95% confidence interval (CI) for progression-free survival and overall survival in 163 patients with stage III–IV oropharyngeal squamous cell carcinoma according to combination of HPV status and LINE-1 methylation

**Additional file 2: Table S2.** Sociodemographic and clinical characteristics in sub-group analyses

**Additional file 3: Table S3.** List of differentially methylated CpG identified comparing relapsed vs not relapsed patients

**Additional file 4: Table S4**. List of differentially expressed genes correlated with differentially methylated CpGs in promoter region identified comparing relapsed versus not relapsed patients

**Additional file 5: Figure S1**. DNA methylation analysis in 5 relapse-free and in 5 relapsed HPV16-negative OPSCC patients. Box plot comparing the methylation level (beta-value) of the non-relapsed (NR) and the relapsed (R) OPSCC patients.

**Additional file 6: Figure S2**. Mapping of the LINE-1 elements. Screenshots from Genome Browser representing the 20 up-regulated genes with hypomethylated promoter region along with “Repeating Elements by RepeatMasker” track.

**Additional file 7: Figure S3**. Mapping of the LINE-1 elements. Screenshots from Genome Browser representing the 20 up-regulated genes with hypomethylated promoter region along with “Repeating Elements by RepeatMasker” track.

**Additional file 8: Figure S4**. Mapping of the LINE-1 elements. Screenshots from Genome Browser representing the 20 up-regulated genes with hypomethylated promoter region along with “Repeating Elements by RepeatMasker” track.

**Additional file 9: Figure S5**. Mapping of the LINE-1 elements. Screenshots from Genome Browser representing the 20 up-regulated genes with hypomethylated promoter region along with “Repeating Elements by RepeatMasker” track.

**Additional file 10: Figure S6**. Mapping of the LINE-1 elements. Screenshots from Genome Browser representing the 20 up-regulated genes with hypomethylated promoter region along with “Repeating Elements by RepeatMasker” track.

**Additional file 11: Figure S7**. Co-expression network of PIK3C2G, GNG4 and NRXN1 based on GeneMANIA. Co-expression: two genes are linked if their expression levels are similar across conditions in a gene expression study.

## Data Availability

The Methylation EPIC raw data and RNA-Seq are publicly available in ArrayExpress repository under accession number E-MTAB-11152 and E-MTAB-12032, respectively. Other data that support the findings of this study are available from the corresponding authors upon request. Reviewers login: Username: Reviewer_E-MTAB-11152, Password: qrzridwu.

## Abbreviations

CI: Confidence interval
CpG: Cytosine–phosphate–guanine
DB: DeltaBeta
FFPE: Formalin-fixed paraffin-embedded
GO: Gene ontology
qMSP: Quantitative methylation-specific PCR
HNSCC: Head and neck squamous cell carcinoma
HPV: Human papillomavirus
HR: Hazard ratio
IHC: Immunohistochemistry
IQR: Interquartile range
LINE-1: Long interspersed nuclear elements 1
OPSCC: Oropharyngeal squamous cell carcinoma
OS: Overall survival
PFS: Progression-free survival
TP53: Tumor protein p53
TSS: Transcriptional start site
