# Supplementary material for: LINE-1 hypomethylation is associated with poor outcomes in locoregionally advanced oropharyngeal cancer
Source: Clin Epigenetics. 2022 Dec 12;14:171. doi: 10.1186/s13148-022-01386-5 (PMC9743592; doi:10.1186/s13148-022-01386-5)
Supplement: Supplementary file 1 — Additional file 1: Table S1. Hazard ratio (HR) and corresponding 95% confidence interval (CI) for progression-free survival and overall survival in 163 patients with stage III–IV oropharyngeal squamous cell carcinoma according to combination of HPV status and LINE-1 methylation [file 13148_2022_1386_MOESM1_ESM.doc]

**Supplementary Table 1**. Hazard ratio (HR) and corresponding 95% confidence interval (CI) for progression-free survival and overall survival in 163 patients with stage III-IV oropharyngeal squamous cell carcinoma according to combination of HPV status and LINE-1 methylation

|  | **Patients** | **Progression-free survival** |  | **Overall survival** |
| --- | --- | --- | --- | --- |
|  |  | **HR (95% CI)^a^** |  | **HR (95% CI)^a^** |
|  |  |  |  |  |
| HPV+ and LINE-1≥55% | 35 | Reference |  | Reference |
| HPV+ and LINE-1<55% | 11 | 2.12 (0.69-6.83) |  | 2.38 (0.76-7.53) |
| HPV- and LINE-1≥55% | 44 | 3.07 (1.42-6.63) |  | 2.87 (1.27-6.47) |
| HPV- and LINE-1<55% | 66 | 4.54 (2.18-9.48) |  | 4.83 (2.24-10.38) |
|  |  |  |  |  |

^a^Estimated from Cox proportional hazards model, adjusted for study, sex, age, T stage, and N stage.
