# Supplementary material for: LINE-1 hypomethylation is associated with poor outcomes in locoregionally advanced oropharyngeal cancer
Source: Clin Epigenetics. 2022 Dec 12;14:171. doi: 10.1186/s13148-022-01386-5 (PMC9743592; doi:10.1186/s13148-022-01386-5)
Supplement: Supplementary file 2 — Additional file 2: Table S2. Sociodemographic and clinical characteristics in sub-group analyses [file 13148_2022_1386_MOESM2_ESM.doc]

**Supplementary Table 2**. Socio-demographic and clinical characteristics in sub-group analyses

|  | **p53 expression analysis**  **(N=89)** | |  | **Methylation analysis**  **(N=10)** | |
| --- | --- | --- | --- | --- | --- |
|  | **N** | **(%)** |  | **N** | **(%)** |
| Sex |  |  |  |  |  |
| Man | 60 | (67.4) |  | 7 | (70.0) |
| Woman | 29 | (32.6) |  | 3 | (30.0) |
| Age (years) |  |  |  |  |  |
| <60 | 24 | (27.0) |  | 4 | (40.0) |
| 60-69 | 32 | (36.0) |  | 3 | (30.0) |
| ≥70 | 33 | (37.0) |  | 3 | (30.0) |
| T stage |  |  |  |  |  |
| T1 | 15 | (16.9) |  | 2 | (20.0) |
| T2 | 15 | (29.2) |  | 4 | (40.0) |
| T3 | 30 | (33.7) |  | 3 | (30.0) |
| T4 | 18 | (20.2) |  | 1 | (10.0) |
| N stage |  |  |  |  |  |
| N0 | 9 | (10.1) |  | 1 | (10.0) |
| N1 | 19 | (21.4) |  | 3 | (30.0) |
| N2 | 54 | (60.7) |  | 6 | (60.0) |
| N3 | 7 | (7.9) |  | 0 | (0.0) |
| Stage (TNM 7^th^ edition) |  |  |  |  |  |
| III | 23 | (25.8) |  | 4 | (40.0) |
| IV | 66 | (74.2) |  | 6 | (60.0) |
| HPV-status |  |  |  |  |  |
| Negative | 64 | (71.9) |  | 10 | (100.0) |
| Positive | 25 | (28.1) |  | 0 | (0.0) |
| Surgery |  |  |  |  |  |
| No | 37 | (41.6) |  | 6 | (60.0) |
| Yes | 52 | (58.4) |  | 4 | (40.0) |
