# Supplementary figures and images for: LINE-1 hypomethylation is associated with poor outcomes in locoregionally advanced oropharyngeal cancer
Source: Clin Epigenetics. 2022 Dec 12;14:171. doi: 10.1186/s13148-022-01386-5 (PMC9743592; doi:10.1186/s13148-022-01386-5)

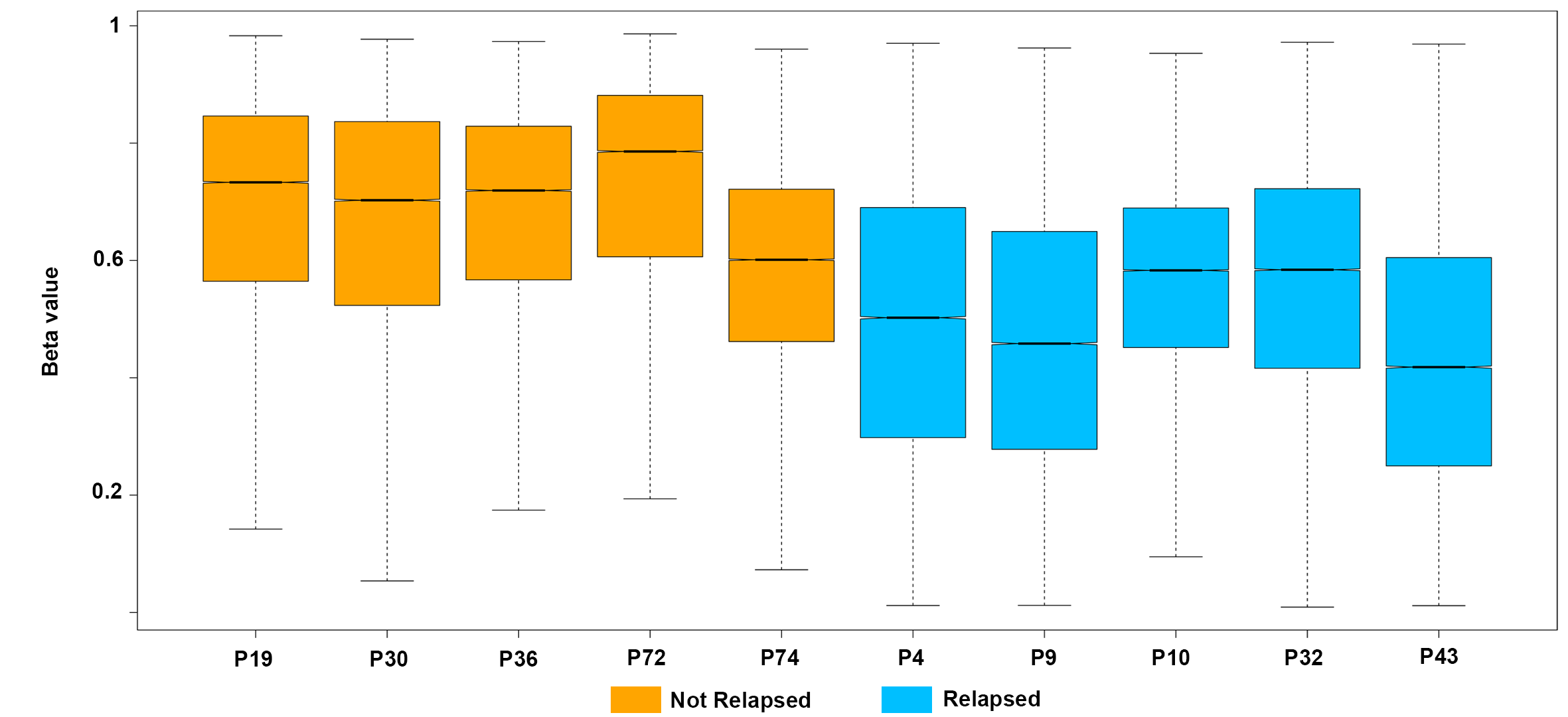

Supplement: Supplementary file 5 — Additional file 5: Figure S1. DNA methylation analysis in 5 relapse-free and in 5 relapsed HPV16-negative OPSCC patients. Box plot comparing the methylation level (beta-value) of the non-relapsed (NR) and the relapsed (R) OPSCC patients. [file 13148_2022_1386_MOESM5_ESM.tif]

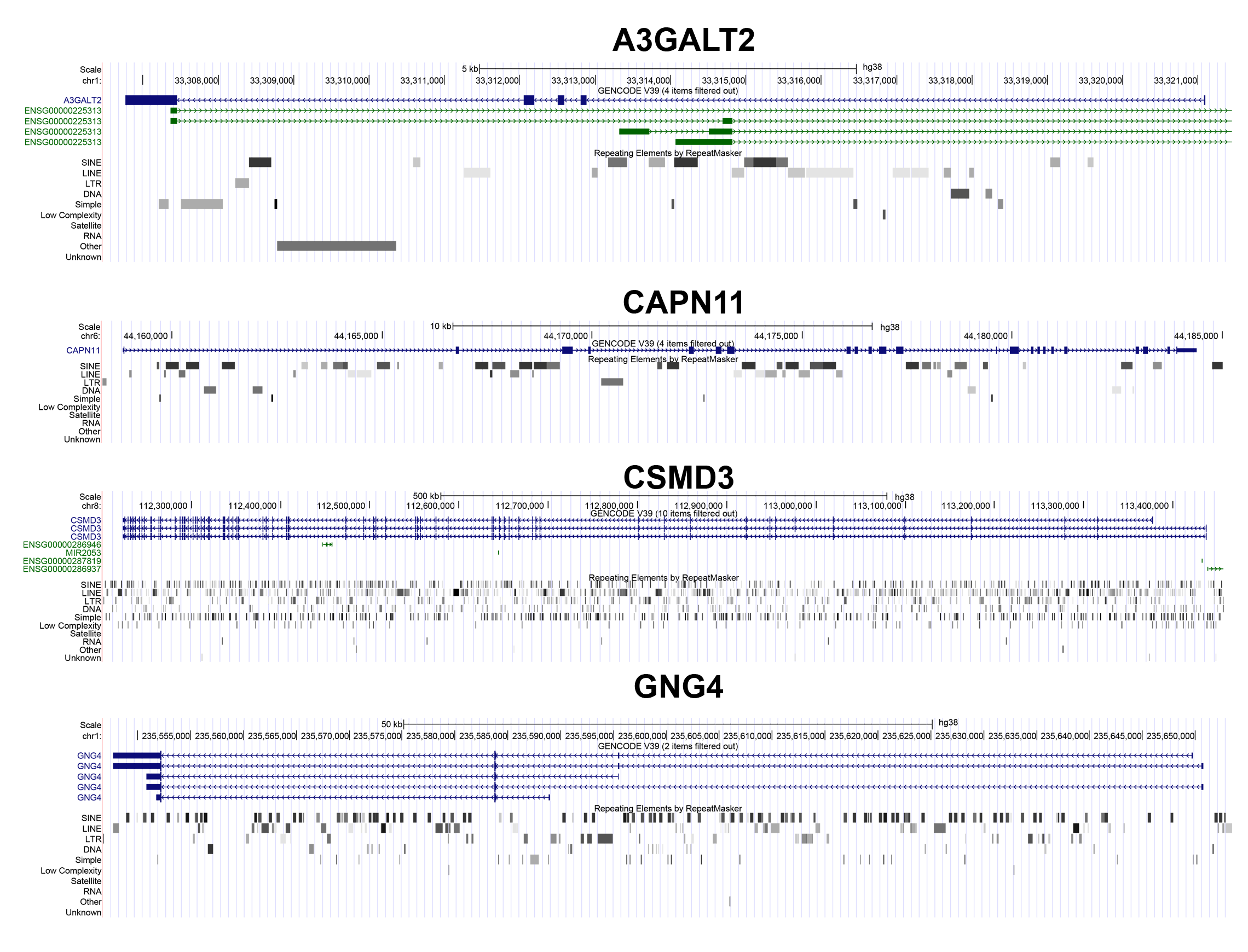

Supplement: Supplementary file 6 — Additional file 6: Figure S2. Mapping of the LINE-1 elements. Screenshots from Genome Browser representing the 20 up-regulated genes with hypomethylated promoter region along with “Repeating Elements by RepeatMasker” track. [file 13148_2022_1386_MOESM6_ESM.tif]

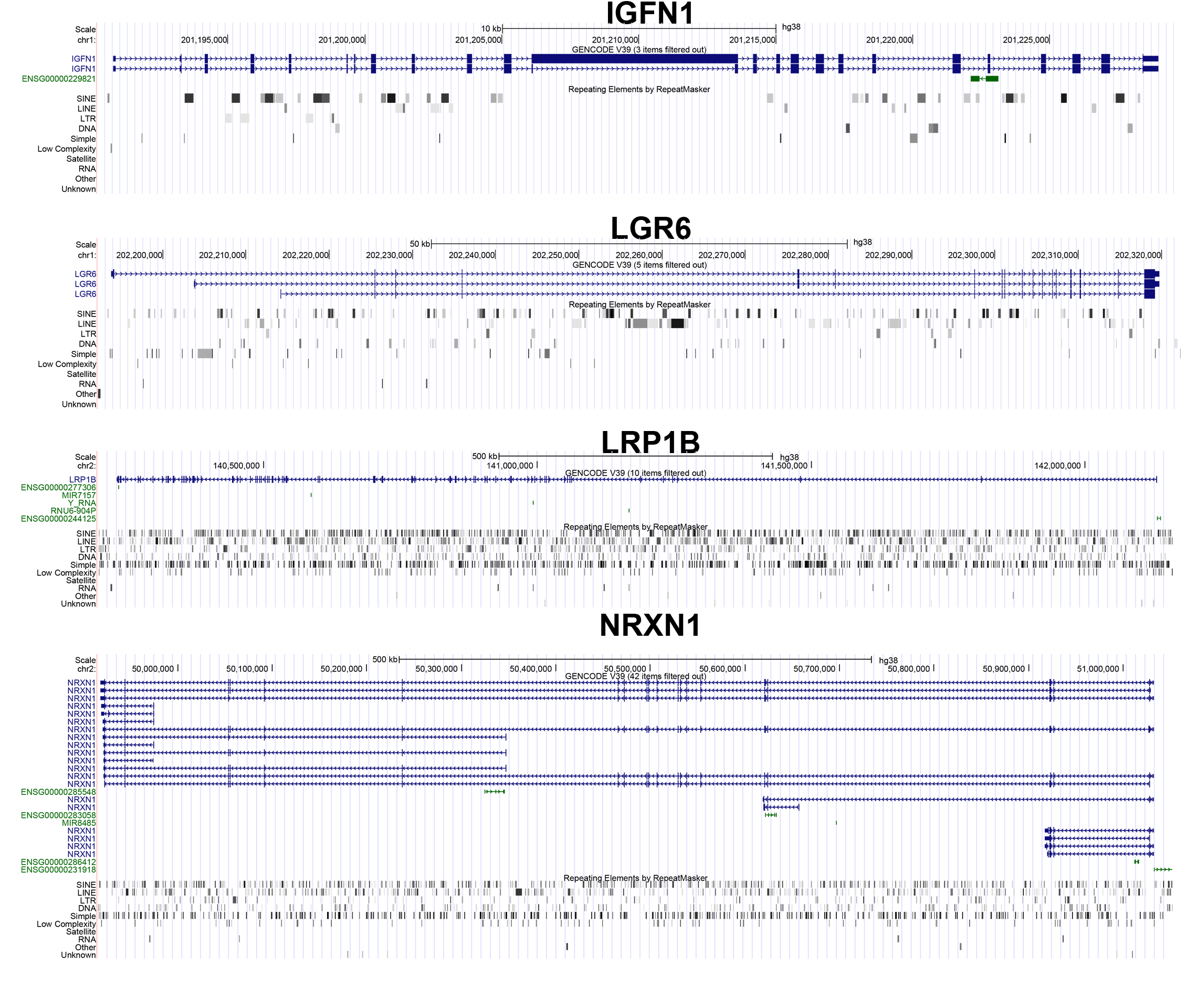

Supplement: Supplementary file 7 — Additional file 7: Figure S3. Mapping of the LINE-1 elements. Screenshots from Genome Browser representing the 20 up-regulated genes with hypomethylated promoter region along with “Repeating Elements by RepeatMasker” track. [file 13148_2022_1386_MOESM7_ESM.tif]

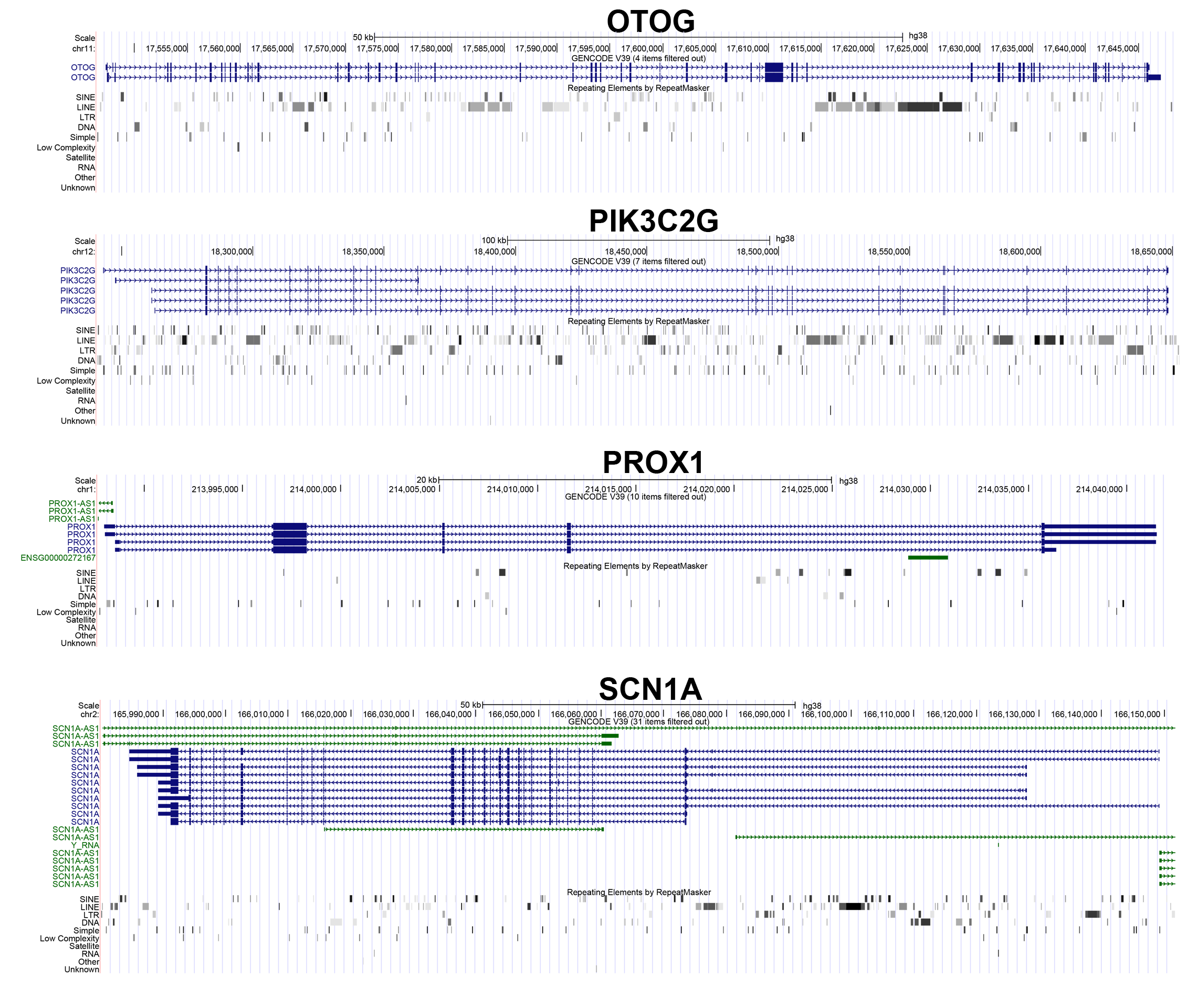

Supplement: Supplementary file 8 — Additional file 8: Figure S4. Mapping of the LINE-1 elements. Screenshots from Genome Browser representing the 20 up-regulated genes with hypomethylated promoter region along with “Repeating Elements by RepeatMasker” track. [file 13148_2022_1386_MOESM8_ESM.tif]

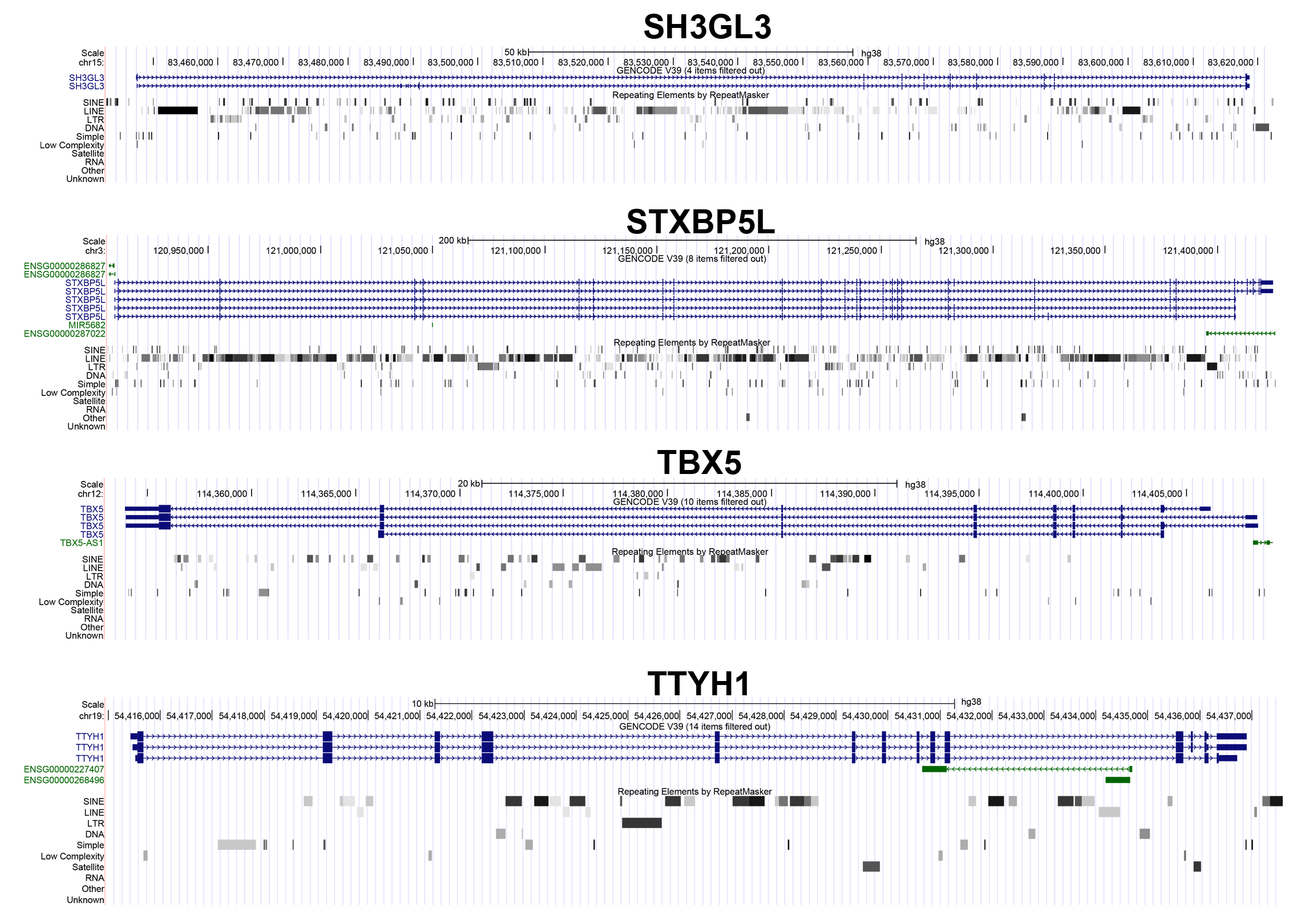

Supplement: Supplementary file 9 — Additional file 9: Figure S5. Mapping of the LINE-1 elements. Screenshots from Genome Browser representing the 20 up-regulated genes with hypomethylated promoter region along with “Repeating Elements by RepeatMasker” track. [file 13148_2022_1386_MOESM9_ESM.tif]

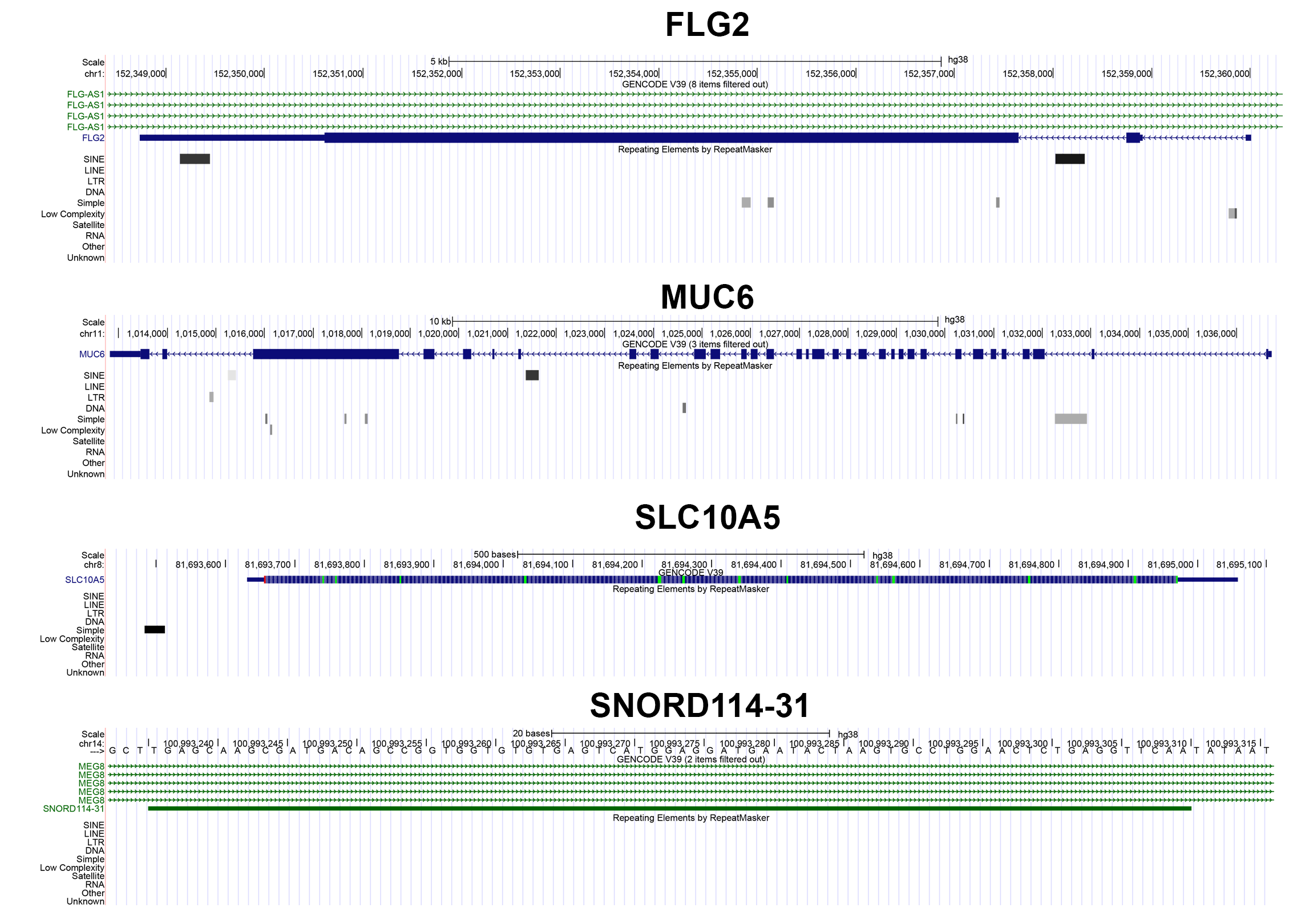

Supplement: Supplementary file 10 — Additional file 10: Figure S6. Mapping of the LINE-1 elements. Screenshots from Genome Browser representing the 20 up-regulated genes with hypomethylated promoter region along with “Repeating Elements by RepeatMasker” track. [file 13148_2022_1386_MOESM10_ESM.tif]

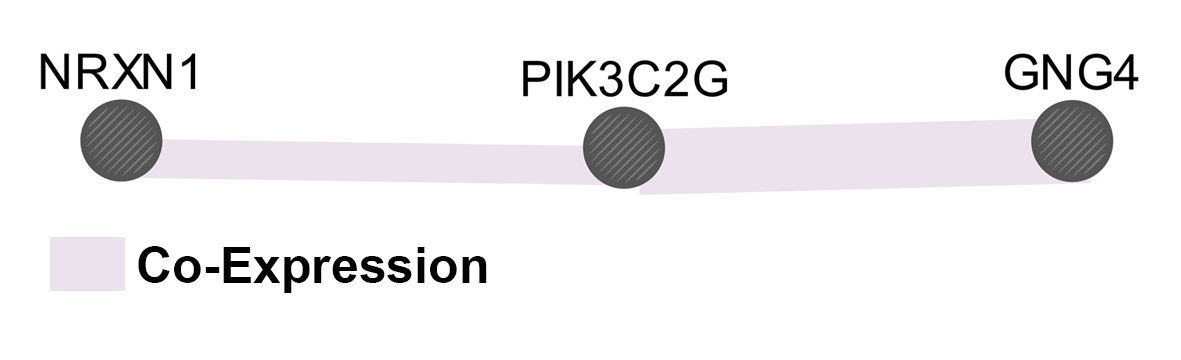

Supplement: Supplementary file 11 — Additional file 11: Figure S7. Co-expression network of PIK3C2G, GNG4 and NRXN1 based on GeneMANIA. Co-expression: two genes are linked if their expression levels are similar across conditions in a gene expression study. [file 13148_2022_1386_MOESM11_ESM.tif]
